# Growth and Distribution of Boron in Oilseed Rape (*Brassica napus* L.) as Affected by Boron Supply

**DOI:** 10.3390/plants11202746

**Published:** 2022-10-17

**Authors:** Anh Quang Dinh, Asif Naeem, Karl H. Mühling

**Affiliations:** Institute for Plant Nutrition and Soil Science, Kiel University, Hermann Rodewald Strasse 2, D-24118 Kiel, Germany

**Keywords:** boron, *Brassica napus*, nutrient solution, translocation factor, B distribution

## Abstract

Oilseed rape (*Brassica napus* L.) is one of the most important oilseed crops. It has relatively high boron (B) requirements for growth. In this study, a hydroponic experiment was performed to determine the critical B requirement and B distribution in *B. napus*. The plants were grown for four weeks at a range of B levels (from 0.25 to 1000 µM) supplied in a nutrient solution. The results showed significant differences in the root and shoot dry matter and B accumulation in these tissues among the supplied B levels. Severe visible symptoms of B deficiency were observed on the leaves at levels lower than 1 µM B and toxicity at 1000 µM B in the nutrient solution. The maximum shoot and root dry matter were recorded at 25 µM B in the nutrient solution. The plants supplied with the lowest and the highest B levels produced 35% and 37% less shoot dry matter than those supplied with 25 µM B, while the corresponding decreases in the root dry matter were 48% and 36%, respectively. The critical concentration of B, which is the lowest concentration at which plants produce 90% of the maximum shoot dry matter, was proven to be 1 µM B for oilseed rape. At this level of external B supply, the B concentration in the shoot was 26.9 mg kg^−1^ DM. It was found that with the increase in B levels in the nutrient solution, the relative distribution of B between the roots and the shoots shifted in favor of the shoots.

## 1. Introduction

Boron (B) is a micronutrient needed for the growth and development of vascular plants [1]. Boric acid (B(OH)_3_), an uncharged molecule, is known to be the major chemical form of B that is taken up by plants [1]. About 96% of B in the soil and in plants exists in the form of boric acid (B(OH)_3_), and only a small fraction occurs as borate anions (B(OH)_4_^−^) [2]. Research has established that B is the only essential element that is taken up by plants as a neutral molecule, i.e., boric acid. In acidic soil, the relative proportion of boric acid over borate anions is higher than that in neutral or alkaline soil, and, hence, the proportional uptake of B will be higher in the former case. Subsequent to its uptake by the roots, B is translocated to the shoots and reproductive structures by a transpiration stream via the vascular tissues [3].

The minimum concentration of a nutrient required in a specified plant part to produce 90% of the maximum biomass or yield of a plant species is called “critical concentration” [4]. The B requirements of plants generally depend on the following two main factors: the plant species and the stage of development [1]. Among the plant species, oilseed plants have the highest B requirements, and the B requirement at the reproductive stages is higher than that at the vegetative stages of growth. Dell and Huang [5] suggested that the apparent higher B requirement during the reproductive stage of growth arises from the fact that the low transpiration rate at this stage reduces the B supply to the reproductive tissues, which are usually located at the far ends of plants.

A number of studies have determined the critical B concentration for oilseed rape, but these vary widely in value and most of them are specific with respect to the sampling time, the plant part sampled and the fraction of B analyzed in the plant tissues. For example, Huang et al. [6] estimated that the critical concentration for B deficiency is 10–14 mg B kg^−1^ of dry matter in the youngest opened leaves and 6–8 mg B kg^−1^ of dry matter in the youngest mature leaves of oilseed rape. On the other hand, [7] reported the critical B concentration to be 32 mg kg^−1^ in the whole shoot and 38 mg kg^−1^ in the mature leaves for *B. napus* grown in calcareous soil. Bergmann [8] observed the typical B deficiency symptoms on the leaves of *B. napus* grown in a nutrient solution containing less than 0.3 µM B. Similarly, Asad et al. [9] used a chelating resin to keep a constant B concentration in the nutrient solution and found that the plants showed a severe decrease in their biomass in a nutrient solution with a B concentration of ≤0.3 µM.

Oilseed rape is a major global oilseed crop that has a high B demand for growth and seed production [10]. Nevertheless, oilseed rape is highly sensitive to B deficiencies, which inhibit the growth of roots and shoots by limiting cell elongation and, in turn, reduce yields [5,11]. To achieve the maximum yield potential, the B requirement for oilseed rape is more than 0.5 mg B kg^−1^ of soil, whereas B toxicity symptoms occur at the soil B level of 5 mg kg^−1^ of soil [12]. In the present study, we investigated the growth and distribution of B in oilseed rape (*Brassica napus*, cv. Alpaga) grown at a range of B levels (0.25–1000 µM B) in a nutrient solution. The relationship between the external B in the nutrient solution and the B concentration in the shoots was developed. The objective of the study was to identify the levels of external B, below and above which growth is limited under the given circumstances, in order to provide guidelines for future experiments in hydroponics on oilseed rape.

## 2. Results

**Figure 1:**
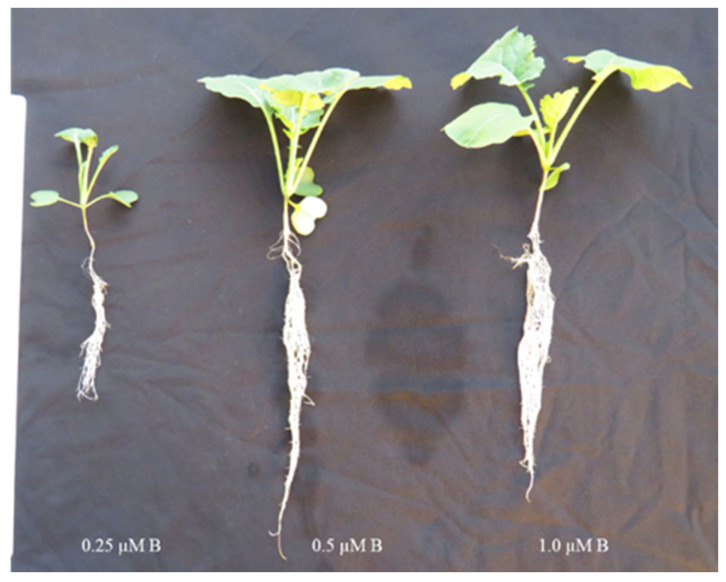
Growth performance of oilseed rape (*Brassica napus*, cv. Alpaga) grown in hydroponic conditions under a low B supply for ten days.

**Figure 2:**
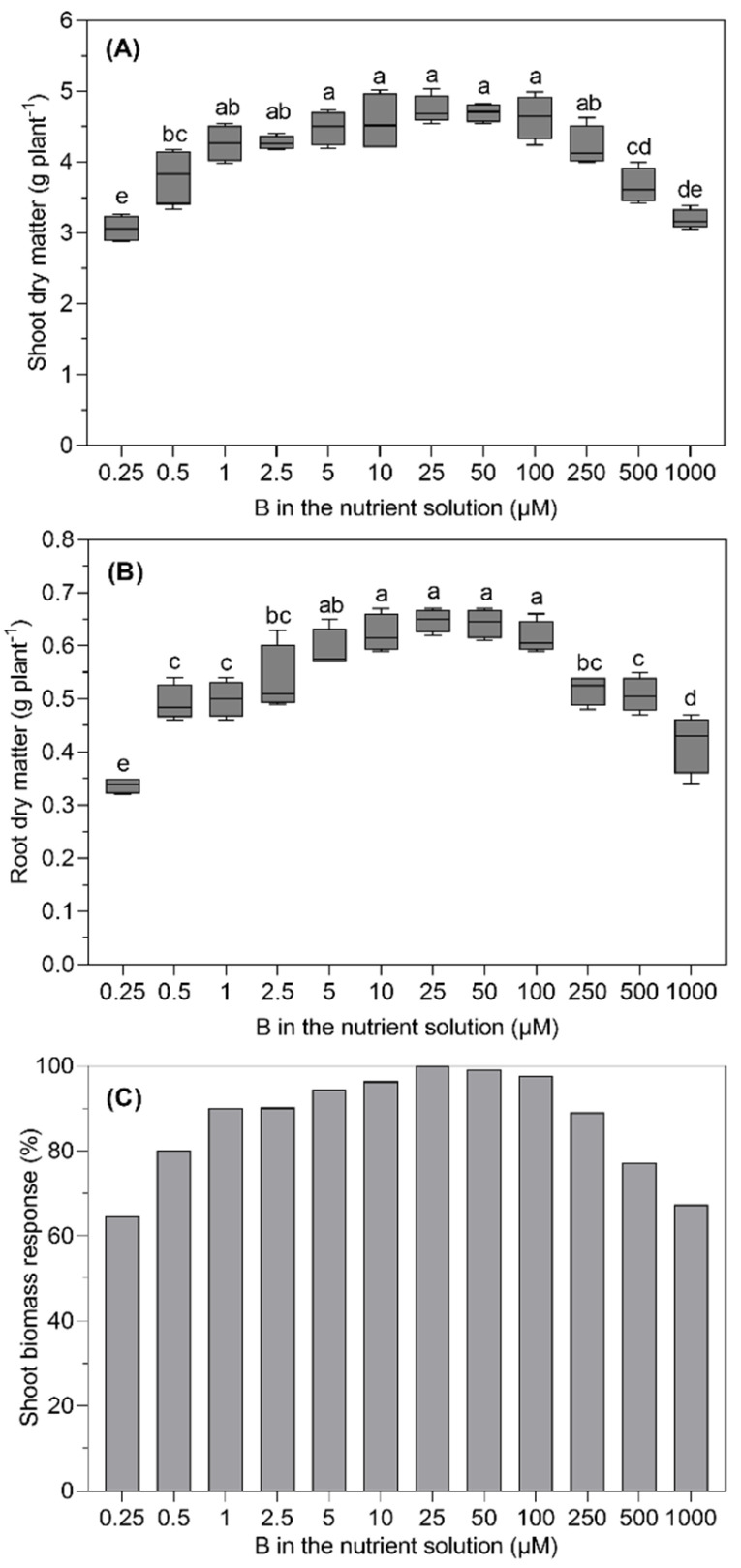
Shoot (**A**) and root (**B**) dry matter yields and shoot biomass response (**C**) of *B. napus* grown with different B concentrations in a nutrient solution for 28 days. The dropbox represents the means (±SE) of four independent pot replicates. The different letters indicate significant differences between the treatments (*p* ≤ 0.05).

Severe visible symptoms of B deficiency and toxicity, such as chlorosis at the leaf margin and leaf tips, were observed on *B. napus* leaves at lower and higher levels of B in the nutrient solution (Figure 1). The root and shoot dry matter of *B. napus* significantly (*p* ≤ 0.05) depended upon the level of B supplied in the nutrient solution (Figure 2). Although non-significant differences in the shoot dry matter were observed in the range from 1.0 to 100 µM B treatments, the dry matter increased with each succeeding level until 25 µM B and started declining at and above 100 µM B in the nutrient solution. With the statistically non-significant difference from that produced at the 10, 50 and 100 µM B treatments, the maximum root dry matter was recorded for the 25 µM B for the treated plants. As compared to the 25 µM B, 48%, 24%, 23% and 18% less root dry matter was produced for the 0.25, 0.5, 1.0 and 2.5 µM B treatments, respectively. Similarly, as compared to the 25 µM B, the higher B levels, such as 250, 500 and 1000 µM, reduced the root dry matter production by 21%, 22% and 36%, respectively.

The low and high B supplies affected the shoot dry matter in a similar way; however, the effect of the lowest or the highest B supply on the shoot dry matter was less severe than that on the root dry matter. The plants supplied with the lowest and highest B levels produced 35% and 37% less shoot dry matter than those achieved with the 25 µM B treatment (Figure 2A). Although the shoot dry matter of the plants treated with the 25 µM B tended to be higher than that produced at the preceding or succeeding levels, the differences were statistically non-significant. Interestingly, the plant treated with 1 µM B in the nutrient solution produced 90% of the maximum shoot dry matter as produced at 25 µM B.

**Figure 3:**
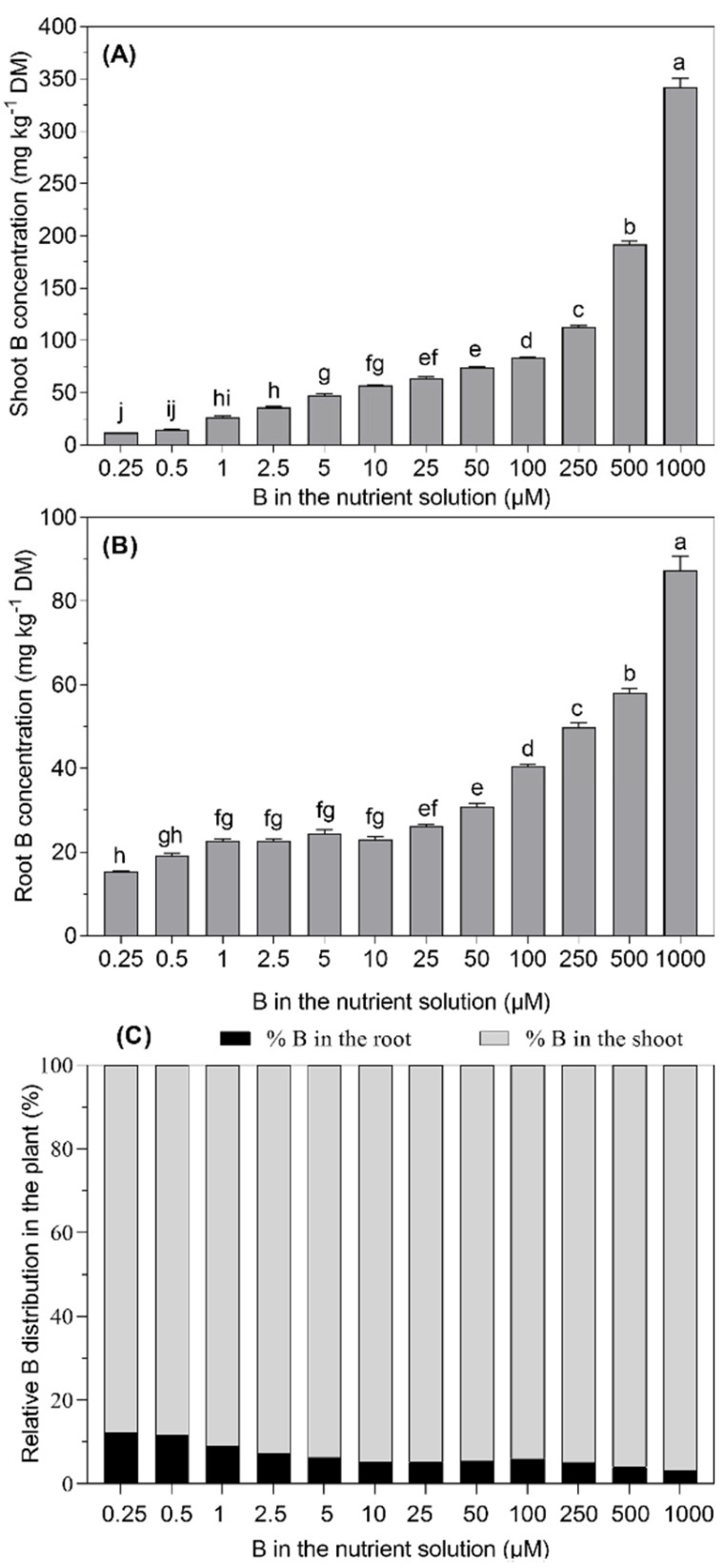
Boron concentration in the shoots (**A**) and roots (**B**) and relative distribution of B in the shoots and roots (**C**)of *B. napus* grown with different B concentrations in a nutrient solution for 28 days. The bars represent the means (±SE) of four independent pot replicates. The different letters indicate significant differences between the treatments (*p* ≤ 0.05).

As expected, the increase in the B supply followed a concomitant increase in the B concentration in the roots and shoots of *B. napus* plants. The lowest B concentration in the roots (15.3 mg kg^−1^ DM) was recorded at 0.25 µM B in the nutrient solution, whereas the increasing B supply from 1.0 to 10 µM B showed a non-significant increase (*p* ≤ 0.05) during the 28-day experimental period. However, the increasing B supply from 25 µM to 100 µM (4-fold) and 1000 µM (40-fold) increased the root B concentration by 1.5-fold and 3.2-fold, respectively. Similarly, the B concentration in the shoots increased significantly with the B supply level in the nutrient solution. The B concentration in the shoots of the plants supplied with the lowest B level in the nutrient solution was 12.1 mg kg^−1^ DM. With the highest B supply, the concentration of B in the shoots was 4-fold higher than that recorded for the 25 µM B treatment. The boron concentration in the shoots was higher than that in the roots for all of the B treatments, and the increasing B supply further favored this trend (Figure 3).

**Figure 4:**
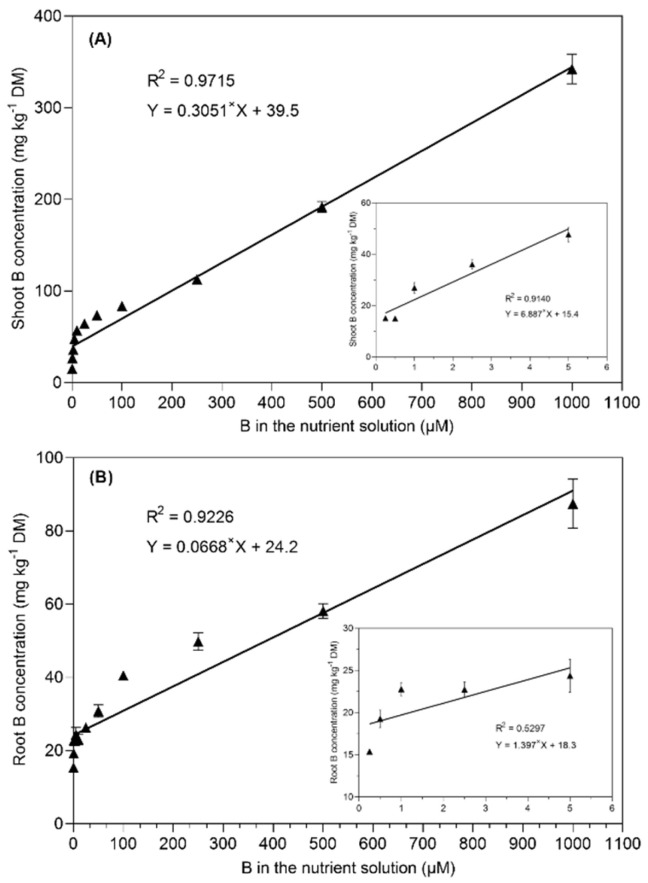
Linear regression between the B concentration in the nutrient solution and the shoot B concentration (**A**) and root B concentration (**B**) in *B. napus* grown with different B concentrations in a nutrient solution for 28 days. The data points represent the means (±SE) of four independent replicates.

The boron translocation from the roots to the shoots was not affected at low B supply levels (from 0.25 to 1 µM B) in the nutrient solution (Table 1). Interestingly, the translocation factor increased with the increasing B concentration in the nutrient solution from 2.5 to 10 µM B and then remained relatively constant until 250 µM B. However, the plants growing under 500 and 1000 µM B in the nutrient solution drastically increased the translocation factor, i.e., it increased by 14% and 22% than in the 25 µM B-treated plants. A highly positive linear relationship (R^2^ = 0.97) was found between the shoot B concentration and the B supply in the nutrient solution after the 28-day experiment (Figure 4). However, a less positive linear relationship (R^2^ = 0.529) was observed between the root B concentration and the increasing B supply from 0.25 to 5 µM B in the nutrient solution.

## 3. Discussion

In this study, we investigated the response of *B. napus* grown at different external B concentrations ranging from deficiency to toxicity in a hydroponic experiment. This is challenging because B has high mobility and is a trace element with a very narrow range between deficiency, adequacy and toxicity. A few studies have been conducted to identify the adequate concentration for *B. napus* growth in hydroponic experiments by using B chelating resins [9,11,13]. With an optimistic expectation, covering a wide range of B supplies, from the lowest (0.25 µM B) to the highest (1000 µM B), the growth response of the plants has been found to follow the optimum curve (Figure 2). Nonetheless, visible symptoms of B toxicity on the leaves of *B. napus* plants at the highest B level (1000 µM B) appeared even before a significant growth retardation was observed. It was found that B deficiency (0.25 µM B) and B toxicity (1000 µM B) severely affected the biomass of *B. napus* (Figure 2) compared to the optimum (25 µM) B supply. This finding is actually in accordance with what was reported by Savic et al. [13], Asad et al. [14] and Yang et al. [15]. The maximum biomass production of *B. napus* plants was recorded at 25 µM B external concentrations (Figure 2). Interestingly, the maximum biomass of *B. napus* obtained at a vegetative growth is slightly higher as compared with what has earlier been reported by Huang et al. [6] at 100 µg L^−1^, Asad et al. [9] at 26.5 µM and Stangoulis et al. [11] at 0.25 mg kg^−1^ of soil. The critical value indicates that *B. napus* plants supplied with different B concentrations responded very well at this concentration; it could be established that the critical B level in the nutrient solution for four-week-old *B. napus* in the present work was found to be 1 µM B. At this B level in the nutrient solution, the B concentration in the shoot was 26.9 mg kg^−1^ DM, which was lower than the critical B concentration (32 mg kg^−1^ DM) in the whole shoots of oilseed rape, as determined by [7]. The reduction in growth and the increase in B concentration in plant tissues as a consequence of B toxicity have been established in *B. napus* [11].

As it was expected, the root and shoot B concentrations increased with increasing the level of B supply in the nutrient solution, always with a higher B concentration in the shoots than in the roots (Figure 3). In 28-day-old *B. napus* plants, the shoot B concentration at the lowest B supply level was 12.1 mg kg^−1^ DM, which is below the critical concentration for a B deficiency in oilseed rape, as described by Savic et al. [16] and Rashid et al. [7].

The observation that the root B concentrations did not significantly increase between the B levels ranging from 2.5 to 10 µM B in the nutrient solution (Figure 3) indicates that the preferential B binding in the cell walls of the roots saturates the cell walls and decreases the ability of the plant to further accumulate B in the roots [17,18]. Nevertheless, the shoot B concentration increased with the increase in the B supply from 2.5 to 10 µM B because only a part of the B is bound to the cell walls of the roots while the remaining free B is translocated to the shoots via a transpiration stream [17,19,20]. The higher B supply levels favored the partitioning of B into the shoots, which accumulated a 3–4-fold higher B than the roots (Figure 2). These findings are in agreement with Dannel et al. [21], who showed that the shoots accumulated higher quantities of B than the roots of sunflowers.

The plants did not show any symptoms of B toxicity during the early stage of vegetative growth; the symptoms became apparent and more severe with time when the plant kept on absorbing and accumulating more B in the leaves [13]. The increase in the root and shoot dry matter of *B. napus* was accompanied by an increase in the B concentration in the plant parts, resulting in an increase in B content in the plant. Altogether, the results are consistent with the view that the preferential B distribution in the shoots of *B. napus* plants is mainly influenced by transpiration, leading to a higher B accumulation in the leaves via the transpiration stream and, finally, resulting in B toxicity.

## 4. Materials and Methods

### 4.1. Plant Culture

The hydroponic experiment was conducted in the greenhouse of the Institute of Plant Nutrition and Soil Science, Kiel University, Germany. The greenhouse conditions were set as follows: 22 °C/18 °C day/night temperature; 60% relative humidity; 14 h d^−1^ photoperiod from 6:00 to 20:00 with a 250–320 μmol photon m^−2^ s^−1^ light intensity recorded by a lightmeter (Li-189, Lincoln, Dearborn, MI, USA). Oilseed rape (*Brassica napus* L. cv. Alpaga) seeds, provided by Norddeutsche Pflanzenzucht Lembke (NPZ), Hohenlieth (Germany), were soaked in an aerated 1 mM CaSO_4_ solution for 24 h. Afterwards, the seeds were rinsed with distilled water and sandwiched between two layers of moist filter paper. The sandwiched seeds were transferred into a container containing a 1 mM CaSO_4_ solution and kept in the dark for 4 days and for 2 days in the light. Six days after germination, the oilseed rape seedlings were transferred into plastic pots containing 4.5 L of aerated, quarter-strength modified Hoagland nutrient solution. The concentration of the nutrient solution was increased to one-half strength on day 4 and to full strength on day 8 after transplanting. The composition of the full-strength nutrient solution was as follows: 2.0 mM Ca(NO_3_)_2_, 0.5 mM K_2_SO_4_, 0.25 mM KH_2_PO_4_, 0.325 mM MgSO_4_, 50 µM NaCl, 2 µM MnSO_4_, 0.4 µM ZnSO_4_, 0.4 µM CuSO_4_, 0.1 µM Na_2_MoO_4_ and 40 µM Fe–EDTA. The B treatments, which were applied from the beginning, included a series of B levels, namely, 0.25, 0.5, 1.0, 2.5, 5.0, 10.0, 25, 50, 100, 250, 500 and 1000 µM B (H_3_BO_3_), in the nutrient solution. The pH of the nutrient solution was adjusted to 6.0–6.5 daily with 0.1 M KOH or HCl throughout the growth period. The nutrient solutions were renewed every 3–4 days, depending on the growth of the plants. To avoid B contamination, double deionized water (18.2 MΩ) was used for the preparation of the nutrient solutions.

### 4.2. Harvesting and Analytical Methods

The experimental treatments, which had four replicates each, were arranged in a completely randomized design. The plants were harvested on day 28 after transplanting, when they had four to five fully expanded leaves. The roots and shoots were harvested separately; they were rinsed with deionized water three times to remove the adhering nutrients from the plant material and dried at 65 °C until they had a constant weight. The dry matter of the roots and shoots was recorded, and the dried tissue samples were ground to a fine powder using a ball mill grinder. Approximately 200 mg of the dried material was digested in 10 mL of 69% HNO_3_ (ROTIPURAN Supra for ICP, 69%) in a closed-vessel 1800 watts microwave digestion system (MARS 6 Xpress, CEM Corporation, Matthews, NC, USA), with the settings described in detail by [22]. Afterwards, the digested samples were diluted with 2% HNO_3_ to 100 mL and stored at 4 °C until further analysis. The concentration of B was determined by an inductively coupled plasma mass spectrometer (ICP-MS; Agilent 7700, Agilent Technologies Inc., Santa Clara, CA, USA). The translocation factor was calculated as the ratio of the B concentration in the shoots to that in the roots [23].

### 4.3. Data Analysis

The significant differences between the treatments were determined by a Duncan’s test and a one-way ANOVA at the level of 0.05 by using the SPSS 22.0 software. All of the values in this study were the means ± SE from four biological replicates. The graphs were created using the GraphPad Prism 8.4.2 software (GraphPad Software Inc., San Diego, CA, USA).

## 5. Conclusions

This study showed that the critical concentration of B in the nutrient solution was 1 µM. At this level, *B. napus* produced 90% of the shoot dry matter response as compared to the sufficient B supply (25 µM). The increasing B supplied in the nutrient solution led to increased shoot and root B concentrations and an increased B distribution in the shoots, which was less than in the roots. The B translocation factor was not affected by the moderate B supply levels in the nutrient solution. There was a close correlation between the B concentration in the nutrient solution and the B concentration in the shoots.

## Figures and Tables

**Table 1:** Distribution of B, ratio of shoot and root dry matter and translocation factor between the shoots and roots of *B. napus* with the B treatments.

B in the Nutrient Solution (µM)	Shoot Total B/Root Total B	Shoot DM/Root DM	Translocation Factor (TF)
0.25	8.92	9.08	0.98
0.5	7.53	7.71	0.97
1	10.16	8.54	1.18
2.5	12.71	7.99	1.59
5	14.80	7.56	1.96
10	18.30	7.34	2.48
25	17.91	7.32	2.45
50	19.30	7.31	2.39
100	13.44	7.53	1.90
250	18.66	8.15	2.27
500	23.94	7.21	3.30
1000	30.12	7.64	3.92

## Data Availability

The data that support the findings of this study are available from the corresponding author upon reasonable request.

## References

[1] Broadley M.B.P., Cakmak I., Rengel Z., Zhao F. (2012). Function of Nutrients: Micronutrients. Marschner’s Mineral Nutrition of Higher Plants.

[2] Wimmer M.A., Goldberg S., Gupta U.C., Barker A., Pilbeam D. (2015). Boron. Handbook of Plant Nutrition.

[3] Brown P.H., Shelp B.J. (1997). Boron mobility in plants. Plant Soil.

[4] Smith F., Reuter D.J., Robinson J.B. (1986). Interpretation of plant analysis: Concepts and principles. Plant Analysis. An Interpretation Manual.

[5] Dell B., Huang L. (1997). Physiological response of plants to low boron. Plant Soil.

[6] Huang L.B., Ye Z.Q., Bell R.W. (1996). The importance of sampling immature leaves for the diagnosis of boron deficiency in oilseed rape (*Brassica napus* cv Eureka). Plant Soil.

[7] Rashid A., Rafique E., Bughio N. (1994). Diagnosing boron deficiency in rapeseed and mustard by plant analysis and soil testing. Commun. Soil Sci. Plant Anal..

[8] Bergmann W. (1992). Nutritional Disorders of Plants: Visual and Analytical Diagnosis.

[9] Asad A., Bell R.W., Dell B., Huang L. (1997). External boron requirements for Canola (*Brassica napus* L.) in boron buffered solution culture. Ann. Bot..

[10] Gupta U.C. (1980). Boron nutrition of crops. Adv. Agron..

[11] Stangoulis J.C., Brown P.H., Bellaloui N., Reid R.J., Graham R.D. (2001). The efficiency of boron utilisation in canola. Funct. Plant Biol..

[12] Shorrocks V.M. (1997). The occurrence and correction of boron deficiency. Plant Soil.

[13] Savic J., Marjanovic-Jeromela A., Glamoclija D., Prodanovic S. (2013). Oilseed rape genotypes response to boron toxicity. Genetika.

[14] Asad A., Blamey F.P.C., Edwards D.G. (2002). Dry matter production and boron concentrations of vegetative and reproductive tissues of canola and sunflower plants grown in nutrient solution. Plant Soil.

[15] Yang L., Zhang Q., Dou J.N., Li L., Guo L.F., Shi L., Xu F.S. (2013). Characteristics of root boron nutrition confer high boron efficiency in *Brassica napus* cultivars. Plant Soil.

[16] Savic J., Romheld V., Nikolic M. (2012). Oilseed rape (*Brassica napus* L.) genotypic variation in response to boron deficiency. Turk. J. Agric. For..

[17] Matoh T. (1997). Boron in plant cell walls. Plant Soil.

[18] O’Neill M.A., Ishii T., Albersheim P., Darvill A.G. (2004). Rhamnogalacturonan II: Structure and function of a borate cross-linked cell wall pectic polysaccharide. Annu. Rev. Plant Biol..

[19] Wimmer M.A., Eichert T. (2013). Review: Mechanisms for boron deficiency-mediated changes in plant water relations. Plant Sci..

[20] Funakawa H., Miwa K. (2015). Synthesis of borate cross-linked rhamnogalacturonan II. Front. Plant Sci..

[21] Dannel F., Pfeffer H., Römheld V. (1998). Compartmentation of boron in roots and leaves of sunflower as affected by boron supply. J. Plant Physiol..

[22] Dinh A.Q., Naeem A., Sagervanshi A., Wimmer M.A., Mühling K.H. (2021). Boron uptake and distribution by oilseed rape (*Brassica napus* L.) as affected by different nitrogen forms under low and high boron supply. Plant Physiol. Biochem..

[23] Wu J., Geilfus C.-M., Pitann B., Mühling K.-H. (2016). Silicon-enhanced oxalate exudation contributes to alleviation of cadmium toxicity in wheat. Environ. Exp. Bot..

